# Recent Advances in Doxorubicin Formulation to Enhance Pharmacokinetics and Tumor Targeting

**DOI:** 10.3390/ph16060802

**Published:** 2023-05-29

**Authors:** Jihoon Lee, Min-Koo Choi, Im-Sook Song

**Affiliations:** 1 BK21 FOUR Community-Based Intelligent Novel Drug Discovery Education Unit, Vessel-Organ Interaction Research Center (VOICE), Research Institute of Pharmaceutical Sciences, College of Pharmacy, Kyungpook National University, Daegu 41566, Republic of Korea; legadema0905@knu.ac.kr; 2 College of Pharmacy, Dankook University, Cheon-an 31116, Republic of Korea; minkoochoi@dankook.ac.kr

**Keywords:** doxorubicin (DOX), formulation strategy, drug resistance, oral formulation

## Abstract

Doxorubicin (DOX), a widely used drug in cancer chemotherapy, induces cell death via multiple intracellular interactions, generating reactive oxygen species and DNA-adducted configurations that induce apoptosis, topoisomerase II inhibition, and histone eviction. Despite its wide therapeutic efficacy in solid tumors, DOX often induces drug resistance and cardiotoxicity. It shows limited intestinal absorption because of low paracellular permeability and P-glycoprotein (P-gp)-mediated efflux. We reviewed various parenteral DOX formulations, such as liposomes, polymeric micelles, polymeric nanoparticles, and polymer-drug conjugates, under clinical use or trials to increase its therapeutic efficacy. To improve the bioavailability of DOX in intravenous and oral cancer treatment, studies have proposed a pH- or redox-sensitive and receptor-targeted system for overcoming DOX resistance and increasing therapeutic efficacy without causing DOX-induced toxicity. Multifunctional formulations of DOX with mucoadhesiveness and increased intestinal permeability through tight-junction modulation and P-gp inhibition have also been used as orally bioavailable DOX in the preclinical stage. The increasing trends of developing oral formulations from intravenous formulations, the application of mucoadhesive technology, permeation-enhancing technology, and pharmacokinetic modulation with functional excipients might facilitate the further development of oral DOX.

## 1. Introduction

Although various options such as immunotherapy and targeted therapy have been developed for cancer treatment, chemotherapy remains an important treatment option for most cancers, especially metastatic cancer [1]. The goal of palliative chemotherapy is to help patients live longer comfortably without the influence of factors that harm their quality of life. This goal can be achieved through the development of oral administration therapy from intravenous chemotherapy. Advantages of oral therapy include noninvasiveness, convenience, and cost-effectiveness; moreover, it may reduce the need for hospital care [2,3].

In addition to providing better quality of life, switching to oral formulations of intravenous drugs may produce beneficial pharmacokinetic profiles. Intravenous injection or infusion contributes to toxicity, which is associated with the high peak plasma concentration of anticancer drugs [4]. In the U.S. pharmaceutical market, approximately 70% of anticancer drugs are oral formulations. However, 40% of drugs in the pipeline and 70% of developing candidate drugs exhibit poor water solubility or low oral bioavailability (BA) [5]. Thus, the development of oral dosage formulations and BA enhancement technology for candidate drugs seems to be at a critical stage [6]. However, there are many obstacles that may impede the development of oral dosage formulations, such as low intestinal solubility and permeability and the high intestinal first-pass effect [7,8,9].

**Figure 1:**
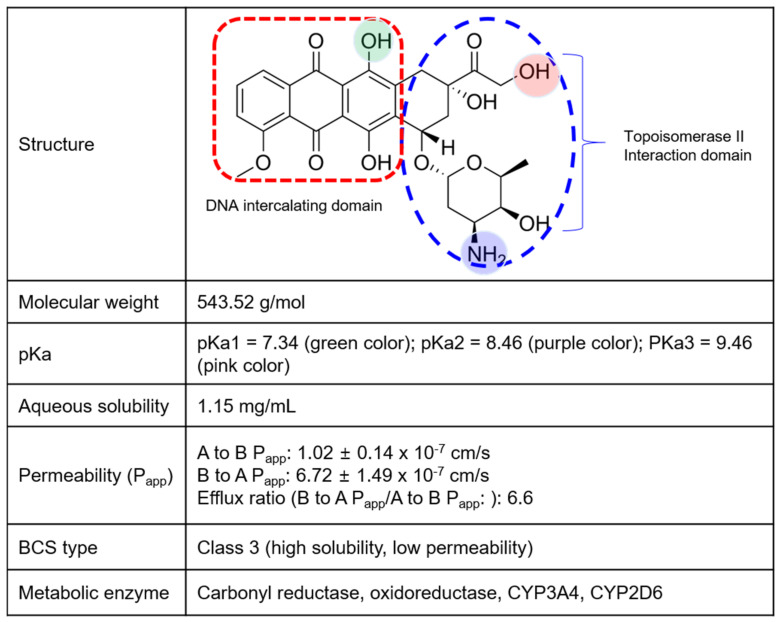
Chemical structure and physicochemical properties of DOX. Data are available at website (https://pubchem.ncbi.nlm.nih.gov/compound/Doxorubicin accessed on 19 May 2023) and reference [19]. P_app_: permeability; A to B: apical to basal; B to A: basal to apical; BCS: biopharmaceutical classification system; CYP: cytochrome P450. Red and blue dotted line indicated DNA intercalating and Topoisomerase II interaction domain.

Doxorubicin (DOX) (Figure 1), an anthracycline drug, is one of the most widely used chemotherapeutic drugs. It is indicated for hematopoietic malignancies and solid tumors and is prescribed to patients with breast and lung cancers, leukemia, and malignant lymphoma [10]. In general, DOX is known to exhibit two mechanisms of action: classical topoisomerase II inhibition and chromatin damage [11]. Topoisomerase II is an enzyme that prevents DNA from excessive or insufficient coiling by creating temporary double-stranded breaks and regenerating DNA via religation [12,13,14]. DOX intercalates into the DNA–topoisomerase II complex through its cyclohexane and sugar moieties and causes DNA damage, followed by p53 pathway-mediated cell cycle arrest [15] (Figure 1). Regarding chromatin damage, the sugar moiety of DOX migrates into DNA, occupying the histone space and causing collapse of the nucleosome [16,17], which leads to cell death [18].

DOX toxicity and therapeutic resistance remain major problems, and resistance to chemotherapeutic drugs can cause treatment failure in >90% of patients with metastatic cancer [20]. DOX resistance may be associated with various mechanisms, including enhanced expression of multidrug resistance (MDR) transporters (such as P-glycoprotein [P-gp], breast cancer resistance protein [BCRP], and MDR-associated proteins [MRPs]), elevated xenobiotic metabolism, increased DNA repair capacity, and increased expression of growth and genetic factors [20,21]. Another obstacle that impedes the development of DOX formulation is its low BA. Limited intestinal permeability as well as the first-pass effect caused by P-gp- and MRP1-mediated efflux of DOX and drug-metabolizing enzymes result in low BA (<5%) [21,22]. DOX is a weakly basic drug and is classified in the biopharmaceutical classification system (BCS) as type 3 because of its high solubility (1.15 mg/mL) and low permeability (6.72 × 10^−7^ cm/s), with an efflux ratio of 6.6 (Figure 1). Therefore, research on oral DOX has mainly focused on enhancing permeability and maintaining the basic pH environment [23].

In this review, we discussed the research on the development of DOX formulations. First, we focused on DOX formulations that have undergone clinical trials and discussed whether they were successful. In addition to the clinical results, we reviewed the obstacles to DOX reformulation with regard to the gastrointestinal environment, drug resistance, and DOX-induced toxicity. Then, we examined the pharmaceutical trials and recent DOX formulations to overcome these obstacles.

## 2. DOX Formulations under Clinical Use or Trials

Remarkable progress has been made in the development of DOX formulations. However, only a few intravenous formulations have been applied as intravenous infusions in the clinical setting. The currently available nanotechnology platforms under clinical use or trials include liposomes, polymeric micelles (PMs), polymeric nanoparticles (PNPs), and polymer–drug conjugates (Table 1).

### 2.1. Liposomes

Liposomes have been used as a drug delivery system for many years since their discovery in 1965 [24]. Their biodegradable characteristics and ability to incorporate hydrophilic, hydrophobic, and amphiphilic drugs allow researchers to encapsulate several drug candidates within liposomes [25,26]. However, in the reticuloendothelial system, high liposome clearance and less effective targeting of liposomes to cancer cells are major obstacles. One of the most well-known modifications involves coating liposome surfaces with polyethylene glycol (PEG), a process known as PEGylation [27]. PEG is a US Food and Drug Administration (FDA)-approved molecule for human administration, which is characterized by nontoxic and nonimmunogenic properties. PEGylation can lead to the formation of a protective hydrophilic layer, can prevent self-aggregation, and can avoid interaction with blood components [28]. Thus, PEGylation reduces complement-mediated lysis by the immune system and prolongs blood circulation times [26,28]. Circulating PEGylated liposomes (PLs) of a size of 100–200 nm are mainly deposited in tumor cells that a have large, leaky spaces in pericytes but not in normal tissue with tight capillary junctions, in what is known as the enhanced permeation and retention (EPR) effect [29,30]. However, PLs block the surface zeta potential, which prevents protein adsorption and may decrease tumor targeting [31]. Decreasing the duration of PEGylation causes liposomes to diffuse out of the lipid membrane system, which are then delivered to the tumor site [32]. Moreover, a specific tumor enzyme that cleaves PEG from liposomes contributes to the detachment of PEG from the liposome system after reaching the target site [33].

In 1995, the first PEGylated liposomal DOX formulation, named Doxil (Janssen Biotech Inc., Horsham, PA, USA), was approved by the US FDA for treating ovarian cancer, Kaposi’s sarcoma, metastatic breast cancer, and multiple myeloma [34]. A similar PEGylated liposomal DOX formulation known as Lipo-dox was approved as a generic version of Doxil by the US FDA in 2012. However, the therapeutic efficacy of Lipo-dox for ovarian cancer was not equivalent to that of Doxil [34]. Caelyx and Zolsketil, two PEGlylated liposomal formulations similar to Doxil, received marketing authorization in 2005 and 2022, respectively, by the European Medicine Agency (EMA) for treating breast and ovarian cancers, multiple myeloma, and Kaposi’s sarcoma [26]. JNS002, a PEGylated liposomal DOX formulation, is under evaluation in a clinical phase III trial involving patients with ovarian cancer, primary fallopian tube cancer, and peritoneal cancer [35]. These formulations are expected to exert EPR effects with reduced cardiotoxicity based on the long circulation time in the blood and reduced distribution to the heart [34] (Table 1).

Phosphatidylcholine/cholesterol liposomes containing citrate (300 mM, pH 4.5) with a size of approximately 150 nm were successfully loaded with DOX with an encapsulation efficiency of over 95%. This formulation, known as Myocet, combined with cyclophosphamide was approved in 2000 by the EMA as a first-line treatment for metastatic breast cancer in adult women [36]. Myocet, a non-PEGylated liposomal DOX formulation, exhibited distinctive pharmacokinetics compared with free DOX and PLs (Doxil and Caelyx). It displayed higher area under the curve (AUC) values than free DOX but lower AUC values than PLs. However, Caelyx, which is characterized by low clearance and long circulating time, could penetrate into skin tissues (e.g., in cases of Kaposi’s sarcoma), which could explain the increased potential to cause hand–foot syndrome, characterized by swelling, pain, and redness on the hands and feet [36]. Hand–foot syndrome is the main effect of the dose-limiting toxicity of PLs, such as Doxil and Caelyx. However, Myocet showed a low incidence of hand–foot syndrome [37].

During the development of active targeting liposomes, a glutathione-conjugated PEGylated liposomal DOX formulation (GSH–PL–DOX) was developed for the delivery of liposomal DOX to the brain through a GSH transporter across the blood–brain barrier. This was confirmed by the 5-fold increase in the delivery of DOX to mice brains compared with that of Doxil [38]. In the phase I/IIa clinical trials, the GSH–PL–DOX formulation was proven to be safe and well tolerated and showed intracranial and extracranial antitumor activity [39]. Human epidermal growth factor receptor 2 (HER2)-targeted antibody anchoring PL–DOX (HER2–PL–DOX) was designed to enhance targeting to HER2-positive advanced breast cancer cells. However, in the phase I clinical trial involving 47 patients with cancer, HER2–PL–DOX failed to show superior beneficial effects [34]. Similarly, epidermal growth factor receptor (EGFR)-targeted antibody (cetuximab) anchoring PL–DOX (EGFR–PL–DOX) targets EGFR-positive breast cancer cells. Patients who received EGFR–PL–DOX did not show hand–foot syndrome, cardiotoxicity, or cumulative toxicity even at the maximum tolerated dose (50 mg/m^2^) [40,41] (Table 1).

### 2.2. PMs

PMs have the advantage of an extremely small particle size (10–100 nm), which makes them efficient for delivering drugs to solid or poorly vascularized tumors. The amphiphilic property allows them to self-assemble in a fluid in vivo after reaching the critical micellar concentration [42]. The structure of a PM entraps a hydrophobic drug in the core, while the hydrophilic shell prevents the removal of the PM via the reticuloendothelial effect, leading to a longer circulation time. This provides PMs an opportunity to accumulate in the tumor site via the EPR effect or actively target the tumor site using a specific ligand [43].

SP1049C is a DOX-loaded PM formulation composed of Pluronic L61 and Pluronic F127 as carrier materials that exert a P-gp inhibitory effect [44,45]. However, its pharmacokinetics and toxic characteristics are similar to those of DOX, and the response ratio of SP1049C in the clinical phase II study was 47% in cases of advanced adenocarcinoma of the esophagus and gastroesophageal junction [46,47,48]. NK911 is a DOX-loaded PEG–polyaspartic acid nanomicelle [49]. In phase II trials in patients with metastatic pancreatic cancer, NK911 was well tolerated and showed a partial response at a dosage of 50 mg/m^2^ every 3 weeks. NK911 showed low plasma concentrations of DOX, suggesting that NK911 is less stable than Doxil [49] (Table 1).

### 2.3. Polymeric Nanoparticles (PNPs)

PNPs are composed of natural ingredients, such as chitosan, dextran, polylactic acid (PLA), polylactide-co-glycolide (PLGA), or polycaprolactone (PCL), with a particle size of 10–1000 nm. PNPs are manufactured as nanocapsules and nanospheres that can entrap drugs within or are associated with a polymer core. They have the advantages of biocompatibility, biodegradability, and design flexibility [50].

Livatag consists of DOX-loaded PNPs formed using alkyl cyanoacrylate (PACA) and covalently linked to squalene. It was developed using Onxeo’s proprietary Transdrug™ technology. Livatag aims to promote the penetration of DOX into tumor cells and enhance the contact between target DNA and DOX, thus bypassing the P-gp-mediated resistance mechanism in tumor cells [51]. It is suitable for the clinical treatment of hepatocellular carcinoma (HCC), and early clinical trials have shown good results. The overall safety and tolerability of Livatag was favorable, with a fully manageable toxicity profile in patients with HCC who had long treatment periods of >1 year. In a clinical phase III trial, although the experimental group did not show the desired effects compared with the high survival rate of the control group that received other anticancer treatments, Livatag as a single agent tended to exhibit a similar level of efficacy as regorafenib [52]. Therefore, the US FDA recently placed Livatag for the treatment of primary liver cancer on a fast-track designation [34] (Table 1).

### 2.4. Polymer-Drug Conjugates

Polymer–drug conjugates can be manufactured by covalently binding a drug to a polymeric carrier. This conjugation confers numerous benefits, including enhanced solubility, controlled drug release, and improved pharmacokinetic drug properties [53]. Clinical evaluation is under way for FCE28068/PK1, N-(2-hydroxypropyl) methacrylamide (HPMA) conjugated to DOX using a Gly–Phe–Leu–Gly peptide spacer [54]. FCE28068/PK1 showed a prolonged plasma circulation time and was mainly cleared by the kidneys without accumulating in the liver [55]. FCE28068/PK1 had no significant cardiotoxicity up to an intravenous dose of 1680 mg/m^2^. It was active against refractory tumors, and the maximum tolerable dose was 320 mg/m^2^, which was 4–5 times higher than the clinical dose of DOX (60–80 mg/m^2^) [55]. In the phase II clinical trial, FCE28068/PK1 showed a considerable response in some patients with breast cancer and non-small cell lung cancer but not in those with colon cancer [56] (Table 1). However, the lack of tissue-targeting ability and biodegradability led to the development of the second formulation.

FCE28069/PK2 is an HPMA polymer–DOX conjugate linked to a galactosamine structure, which binds to the hepatic asialoglycoprotein receptor. It was designed for treating primary liver cancer. When FCE28069/PK2 was intravenously administered to patients with primary or metastatic liver cancer, liver-specific DOX delivery could be achieved, and some patients showed a partial response. However, a patient who received FCE28069/PK2 at a dose of 160 mg/m^2^ had grade 4 neutropenia and grade 3 mucositis, and DOX targeted to the liver was generally distributed to normal liver cells rather than to cancer cells. In other words, 16% of the dose was distributed to the liver but only 3% was distributed to tumor cells [57] (Table 1).

**Table 1:** DOX formulations under clinical use or trials.

Carrier Type	Formulation and Route of Administration	Name	Clinical Results	References
Liposomes	PEGylated liposome (PL), IV	Doxil, approved by the FDA	EPR Treatment of ovarian cancer, Kaposi’s sarcoma, metastatic breast cancer, and multiple myeloma	[34]
	PL, IV	Lipo-dox, approved by the FDA	EPR Therapeutic efficacy was not equivalent to that of Doxil in patients with ovarian cancer	[34]
	PL, IV	Caelyx, approved by the EMA	EPR Treatment of ovarian cancer, Kaposi’s sarcoma, metastatic breast cancer, and multiple myeloma	[26]
	PL, IV	Zolsketil, approved by the EMA	EPR Treatment of ovarian cancer, Kaposi’s sarcoma, metastatic breast cancer, and multiple myeloma	[26]
	PL, IV	JNS002 Phase III	EPR Treatment of ovarian cancer, primary fallopian tube cancer, and peritoneal cancer	[35]
	Non-PEGylated liposome, IV	Myocet, approved by the EMA	EPR A first-line treatment for metastatic breast cancer in adult women, in combination with cyclophosphamide	[36]
	Glutathione-conjugated PL, IV	GSH–PL–DOX Phase I/IIa	Brain targeting through a GSH transporter across the blood–brain barrier Safe and well tolerated with intracranial and extracranial antitumor activity	[39]
	HER2-targeted antibody anchoring PL, IV	HER2–PL–DOX Phase I	HER2-targeting Failed to provide beneficial effect superior to Doxil in patients with breast cancer	[34]
	EGFR-targeted antibody anchoring PL, IV	EGFR–PL–DOX Phase I	EGFR-targeting At 50 mg/m^2^, hand–foot syndrome, cardiotoxicity, or cumulative toxicity did not occur in any patient with glioblastoma and breast cancer	[40,41]
PMs	PM with two nonionic pluronic block copolymers, IV	SP1049C Phase III	Inhibition of P-gp-mediated DOX efflux In a phase II trial involving 21 patients, 9 patients had a partial response and 8 patients had a minor response. The overall response rate was 47%	[46,47]
	PEG–polyaspartic acid nanomicelle, IV	NK911 Phase II	EPR Well tolerated at 50 mg/m^2^ in 23 metastatic pancreatic cancer patients, and a partial response was achieved	[49]
PNPs	Polyalkyl cyanoacrylate (PACA) nanoparticle, IV	Livatag Phase III	The phase III clinical trial did not achieve the desired effects in patients with advanced hepatocellular carcinoma	[51,52]
Polymer–drug conjugates	HPMA copolymer–GFLG–DOX, IV	FCE28069/PK1 Phase II	EPR and pinocytosis The maximum tolerable dose was 320 mg/m^2^, and no polymer-related toxicities were observed A considerable response occurred in some patients with breast and non-small cell lung cancer, but no response was noted in colorectal cancer patients Lack of biodegradability of the polymer main chain	[54,55,56]
	HPMA copolymer–GFLG–DOX–galactosamine, IV	FCE28069/PK2 Phase II	Galactosamine-mediated uptake Liver-specific delivery using galactosamine-modified polymers, and a partial response was achieved in patients with liver cancer Grade 4 neutropenia and grade 3 mucositis	[57]

PL: PEGylated liposome; PM: polymeric micelle; PNP: polymeric nanoparticle; IV: intravenous injection; EPR: enhanced permeation and retention; FDA: Food and Drug Administration; EMA: European Medicine Agency; HPMA: N-(2-hydroxypropyl) methacrylamide; GFLG: glycyl-phenylalanyl-leucyl-glycine.

## 3. Obstacles in and Strategies for Formulating DOX to Enhance Oral BA and Tumor Targeting

As mentioned previously, all DOX formulations in clinical applications are currently administered intravenously. Therefore, many studies on anticancer drugs have focused on the development of oral formulations. Pharmaceutical and chemical strategies have been employed to increase chemical stability in gastrointestinal fluids, increase aqueous solubility, and reduce the first-pass effect. An oral formulation of trifluridine in combination with tipiracil, an inhibitor of thymidine phosphorylase, increased the oral BA of trifluridine by 38-fold and has been approved by the US FDA, the EMA, and the Ministry of Health, Labour and Welfare of Japan (MHLW) [58]. Successful oral formulations of Tegafur from intravenous 5-fluorouracil have been approved by the EMA and MHLW using a prodrug approach in combination with uracil, which acts as a metabolic inhibitor of dihydropyrimidine dehydrogenase [58].

**Figure 2:**
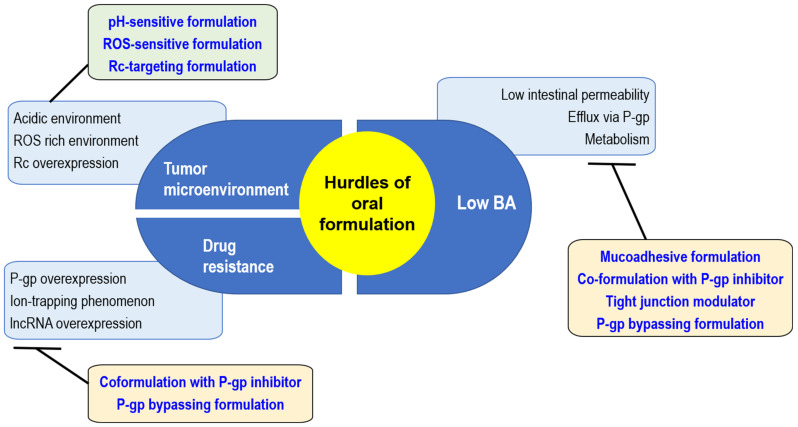
Challenges in the development of DOX oral formulations and the corresponding formulation strategies. ROS: reactive oxygen species; Rc: Receptor; P-gp: P-glycoprotein; lncRNA: long noncoding RNA; BA: bioavailability.

Oral anticancer drugs are effective and convenient for patients. Knowledge regarding the pharmacokinetics-altering strategies of other drugs will guide DOX oral formulation. Therefore, we reviewed these challenges for oral DOX formulations in terms of pharmacokinetic obstacles as well as the occurrence of DOX resistance and the effort to overcome these limitations using a DOX formulation strategy (Figure 2).

### 3.1. Formulation Strategy Based on Tumor Microenvironments for the Targetability of DOX in the Preclinical Stage

#### 3.1.1. pH-Sensitive Formulation

Deregulated energy metabolism, insufficient perfusion, and uncontrolled proliferation collectively confer particular characteristics to the tumor microenvironment, including acidity, hypoxia, increased lactate concentrations, and reduced glucose concentrations [59]. Although the pH of the interstitial space of solid tumors ranges from slightly acidic to neutral (pH 6.4–7.0), the central regions of solid tumors are intensely acidic because of reduced O_2_ and glucose concentrations and, correspondingly, increased H^+^ and lactate concentrations that are observed with increasing distance from the vascular system [59].

To enhance drug release into tumor tissues, pH-sensitive liposomes are currently being researched. The most popular base lipid for fabricating pH-sensitive liposomes is phosphatidylethanolamine (PE). However, PE cannot form a stable liposome on its own; therefore, additional amphiphilic molecules are added as stabilizers. At physiological pH, these stabilizers are inserted between PE molecules in an ionized form, leading to the production of stable liposomes. In an acidic environment, however, protonated stabilizers cause the reversion of PE molecules and disrupt liposomes, leading to a burst release of inner contents, including DOX [60,61,62].

De Oliveira Silva et al. [63] synthesized a formulation of cholesteryl hemisuccinate (CHEMS) and distearoyl PE polyethyleneglycol2000 (DSPE-PEG2000) in a dioleoyl-phosphatidyl-ethanolamine (DOPE)-based pH-sensitive liposome (DOPE:CHEMS:DOPE-PEG2000 = 5.8:3.7:0.5) with a size distribution of 125–135 nm. This formulation showed higher uptake by tumors than by control tissue and higher specificity for tumors in 4T1 tumor-bearing mice. Moreover, the researchers continued the study in healthy mice to monitor acute cardiotoxicity and other side effects. Compared with normal DOX, this pH-sensitive liposome seemed to be more effective and safe, as indicated by the 2-fold reduction in QT interval prolongation on an electrocardiogram in the pH-sensitive liposome treatment group [64] (Table 2).

Bobde et al. [65] conjugated DOX and poly N-(2 hydroxypropyl) methacrylamide via hydrazone bonding and developed pH-sensitive PNP, i.e., HPMA–NH-DOX, which releases DOX 5 times faster in an acidic intratumor environment (pH 6.5). Further, a faster release is observed in a more acidic tumor environment (pH 5.5) in MCF-7 and 4T1 cell lines (Table 2).

pH-sensitive micelles composed of DSPE-PEG2000 and oleic acid at a ratio of 10:6 with a size of 12.8 nm and a zeta potential of −2.7 mV were prepared, and DOX was incorporated with a loading efficiency of 92% [66]. A mixture of DSPE-PEG2000 and oleic acid was self-assembled, wherein the hydrophobic DSPE constituted the inner core with DOX, and PEG2000, as a nontoxic hydrophilic polymer, produced a hydrophilic shell to provide steric stability and protection from opsonization. Oleic acid acts as a pH-sensitive indicator, and DOX incorporated into this micelle was released faster at pH 5.0 than at pH 7.4. As a result, pH-sensitive micelles showed a 7-fold tumor shrinkage in 4T1 tumor-bearing mice compared with free DOX when administered intravenously (5 mg/kg/day, every other day, four times) [66] (Table 2).

#### 3.1.2. Reactive Oxygen Species (ROS)-Sensitive Formulation

Cancer initiation and progression can slightly increase ROS levels. Therefore, cancer cells thrive on moderately higher ROS levels than normal cells because they have developed stronger antioxidant systems. This feature renders cancer cells more sensitive to external stimuli that further increase ROS production [67]. An increasing number of therapeutic strategies are currently being developed to elevate ROS levels and overwhelm the redox adaptation of the same cells as well as ROS-responsive formulations containing anticancer therapeutics. Here, we summarized several DOX formulations that initiate burst release in response to elevated ROS levels in tumor cells.

ROS-sensitive liposomes using 10,10′-diselanediylbis decanoic acid (DDA) as a fundamental building block of various ratios of egg l-α-phosphatidylcholine (egg PC), DOPE, and 1,2-dioleoyl-sn-glycero-3-phosphocholine were prepared and characterized. The optimum formulation of DOPE/egg PC/DDA at a molar ratio of 37.5/60/2.5% showed a 30% burst release in 0.1% H_2_O_2_ at pH 6.5 through diselenide bond cleavage induced by the ROS signal. Intravenous injection of this redox-sensitive formulation containing DOX into C26-tumor-bearing mice showed a 40-fold higher AUC than that of free DOX, efficiently suppressed C26 tumor growth, and improved the distribution of DOX in tumor cells [68] (Table 2).

pH- and ROS-sensitive mesoporous silica nanoparticles (MSNs) with surface modifications using chitosan–folate conjugate (DOX–MSN-SS-CH-FA) have been developed for breast cancer therapy [69]. DOX release significantly increased in the presence of 10 mM GSH and at pH 5.5, suggesting a dual responsive (pH and ROS) formulation. DOX–MSN-SS-CH-FA was activated by ROS and acidic pH and was engulfed by the tumor via the folate receptor to release DOX into tumor cells following its intravenous injection in Ehrlich ascites carcinoma (EAC)-bearing BALB/c mice. DOX–MSN-CH-FA prolonged survival in EAC tumor-bearing mice with a decrease in tumor volume; however, the cardiotoxicity markers remained unchanged [69] (Table 2).

As mentioned previously, ROS-responsive formulations resulted in a positive therapeutic response by enhancing the targetability of the DOX formulation. Therefore, decreasing the ROS level might suppress the DOX response (Figure 2). Nuclear factor erythroid 2-related factor 2 (Nrf2) is a regulator gene that protects cells from oxidative stress and is known to play a key role in cancer progression [70,71]. Ryoo et al. [72] reported the upregulation of Nrf2 expression in DOX-resistant cancer cells, which resulted in reduced ROS levels in cancer cells, leading to decreased DOX efficacy. Moreover, cancer cells with low ROS levels have a tendency to express more P-gp in the cell membrane, as demonstrated by the positive association between Nrf2 and P-gp expression; accordingly, most DOX-resistant cancer cells showed Nrf2 overexpression [70,72]. To interfere with Nrf2 action using short hairpin RNA (shRNA) or small interfering RNA (siRNA) as a strategy to overcome drug resistance, Gu et al. [73] developed hyaluronidase-responsive multilayer liposomes (HLCNs) with cisplatin and Nrf2 siRNA. In vivo results revealed a 4-fold increase in cytotoxicity. Additionally, in mice with xenograft osteosarcoma, HLCN showed a 2-fold decrease in tumor volume with a low cytotoxic effect. This formulation showed favorable and sustained biodistribution of cisplatin in tumor tissues along with its rapid elimination in other organs.

#### 3.1.3. Receptor (Rc)-Targeted Formulation

Prolonged blood circulating formulations are likely to accumulate in tumor tissue with an EPR effect due to the leakiness of tumor vasculature and poor lymphatic drainage [74]. Some receptors are overexpressed on the surface of tumor cells, which could guide targeted drug therapy. For example, formulations modified with tumor-targeting molecules such as folate and transferrin can be easily recognized and internalized by tumor cells due to their overexpression of folate or transferrin receptors [75]. Hyaluronic acid (HA) is an important linear polysaccharide component that exists in the extra-cellular matrix and has been reported with high specific affinity to CD44 receptors on cell surfaces [76], which broadened its application as a tumor targeting delivery system.

Wang et al. [77] prepared folate conjugated PEG-PLGA micelles containing DOX with or without SIS3, a potent P-gp and BCRP inhibitor (i.e., FA/DOX and FA/DOX/SIS3, respectively). Sustained release of DOX from both FA/DOX and FA/DOX/SIS3 micelles could be maintained for more than 48 h and both formulations significantly increased AUC and decreased the elimination half-life of DOX following IV injection compared with free DOX but showed comparable pharmacokinetic behavior between FA/DOX and FA/DOX/SIS3 micelles. In addition, FA/DOX showed a significantly higher intratumor DOX concentration and increased cytotoxicity in MCF-7 cells. By co-encapsulation of SIS3 (FA/DOX/SIS3), DOX concentration in tumor tissue significantly increased compared with FA/DOX in MCF-7/ADR cells. In addition, FA/DOX/SIS3 reduced tumor size more effectively and prolonged survival rate in MCF-7/ADR bearing BALB/c nude mice compared with FA/DOX treatment. Collectively, the results suggested the contribution of the folate targeted formulation and the co-delivery of the efflux pump inhibitor SIS3 to the better therapeutic efficacy and the reversal of DOX resistance [77] (Table 2).

Qiu et al. [78] prepared pH-sensitive and tumor targeting HA-2-(octadecyloxy)-1,3-dioxan-5-amine (HOD) PM and incorporated an acid-sensitive DOX-NN-VES prodrug (i.e., a conjugate of DOX and vitamin E succinate (VES) using a hydrazone bond (NN)). The pH-sensitive HOD PMs are internalized by CD44-mediated endocytosis via HA conjugates. Inside the tumor cells, HOD polymers are depolymerized to release the prodrug DOX-NN-VES. The hydrazone bond of DOX-NN-VES is also rapidly broken in the acidic environment to release the free DOX and VES. VES can inhibit the P-gp efflux pump to increase the accumulation of DOX in MCF-7/ADR cells. Therefore, this formulation can display higher efficiency in overcoming DOX resistance. In MCF-7/ADR tumor bearing mice, the HOD PM enclosed DOX-NN-VES prodrug reduced tumor weight by 2.28-fold, accompanied by reduced cardiotoxic side effect [78].

Scheeren et al. [79] proposed transferrin (Tf) and poloxamer-integrated pH-sensitive PLGA nanoparticles (Tf–DOX–PLGA) to bypass the P-gp-mediated DOX efflux. During the cell cycle, dividing cells show high expression of the transferrin receptor (TfR) for iron intake. TfR has the ability to uptake molecules via TF-mediated endocytosis, and the Tf-incorporated formulation could be uptaken by TfR. The engulfed Tf–DOX–PLGA nanoparticles release DOX and poloxamer into the cells. Poloxamer has been reported to have multiple functions in P-gp overexpressed cells, such as inhibiting P-gp and depleting ATP in mitochondria, resulting in ROS generation and cytochrome c release, which lead to apoptosis [80]. Tf–DOX–PLGA treatment in DOX-resistant NCI/ADR ovarian tumor cells showed a significant decrease in cell viability, from 80% to 20%, compared with free DOX treatment. In addition, the results of a cell cycle arrest study showed that most cells affected by Tf–DOX–PLGA treatment were arrested in the G1 phase, with a 2-fold increase in apoptotic cell death [79] (Table 2).

**Table 2:** Formulation strategy using tumor microenvironments for tumor targetability of DOX in preclinical stage.

Carrier–Type	Formulation & Route of Administration	Experimental Research	Findings	References
pH-sensitive PLs	DOX-loaded PL (DOPE: CHEMS: DOPE-PEG2000 = 5.8:3.7:0.5), IV	4T1 tumor-bearing mice	Long circulating pH-sensitive liposome. Higher tumor uptake in 4T1 tumor-bearing mice	[63]
		Healthy mice	Less QT interval prolongation on an electrocardiogram (reduced cardiotoxicity)	[64]
pH-sensitive PNPs	DOX and pHPMA conjugates via hydrazone bond (HPMA-NH-DOX), IV	4T1, MCF-7 cell	A 5-fold faster DOX release in acidic intratumor (pH 6.5) and intratumor cellular (pH 5.5) environments than at pH 7.4	[65]
pH-sensitive PMs	DOX-loaded micelle (DSPE-PEG2000/OA = 10:6), IV	4T1 tumor-bearing mouse	pH-sensitive DOX release, 7-fold tumor shrinkage	[66]
ROS-sensitive liposomes	DOPE/Egg PC/DDA = 37.5/60/2.5%, IV	Walker 256 carcinosarcoma-bearing rat	A 3-fold faster DOX release at pH 5.0 than at pH 7.4 A 3-fold higher apoptosis rate	[68]
pH- and ROS-sensitive MSNs	Chitosan-folate conjugated MSN (DOX-MSN-SS-CH-FA), IV	C26-tumor-bearing mice	A 30% burst release in 0.1% H_2_O_2_ at pH 6.5 through the diselenide bond cleavage induced by the ROS signal The DOX-loaded liposome showed a 40-fold higher AUC than free DOX, efficient suppression of C26 tumor growth, and improved DOX distribution in tumors	[69]
Rc-targeted PMs	Folate targeted PM co-delivery of DOX and SIS3 (FA/DOX/SIS3), IV	SD rat	EPR and folate Rc-mediated endocytosis P-gp and BCRP inhibition by SIS3 6.1-fold increased AUC and 3.9-fold decreased clearance of DOX compared with free DOX	[77]
	FA/DOX/SIS3, unilateral axillary injection	MCF-7/ADR bearing nude mice	EPR and folate Rc-mediated endocytosis P-gp and BCRP inhibition by SIS3 Increased DOX accumulation in tumor tissue Inhibited tumor growth and prolonged the lifetime in DOX resistant tumor mice	
Rc-targeted and pH-sensitive PMs	HOD PM enclosed DOX-NN-VES, IV	MCF-7/ADR bearing nude mice	EPR and CD44-mediated endocytosis pH-sensitive DOX release at acidic intratumor organelles by hydrazone bond cleavage Increased DOX accumulation in tumor tissue Increased apoptosis and 2.28-fold decreased tumor weight compared with free DOX	[78]
Rc-targeted and pH-sensitive PNPs	Transferrin (Tf)- and poloxamer-integrated pH-sensitive PLGA NPs (Tf–DOX–PLGA), IV	NCI/ADR ovarian tumor cells	P-gp inhibition in tumor cells Significant decrease in cell viability from 80% to 20% compared with free DOX Arrested cell cycle in the G1 phase and increased apoptotic cell death by 2-fold	[79]

PL: PEGylated liposome; PNP: polymeric nanoparticles; PM: Polymeric micelles; MSN: mesoporous silicate nanoparticles; IV: intravenous injection; Rc: Receptor; CHEMS: cholesteryl hemi succinate; DSPE-PEG2000: distearoyl phosphatidyl ethanolamine polyethyleneglycol2000; DOPE: dioleoyl phosphatidyl ethanolamine; pHPMA: Poly N-(2 hydroxypropyl) methacrylamide; OA: oleic acid; ROS: reactive oxygen species; AUC: area under the curve; HOD PM enclosed DOX-NN-VES: HA-2-(octadecyloxy)-1,3-dioxan-5-amine (HOD) PM incorporating a conjugate of DOX and vitamin E succinate using a hydrazone bond (DOX-NN-VES).

### 3.2. Formulation Strategy for Overcoming DOX Resistance in the Preclinical Stage

#### 3.2.1. Overexpression of P-gp in Tumor Cells

P-gp is found not only in the gastrointestinal tract but also in tissues associated with various other cancers, especially melanoma and central nervous system cancer, with extremely high expression in renal and colon cancers [81]. Unexpectedly, DOX could induce P-gp expression in cancer cell membranes; it shows significant correlation with increased P-gp expression in cancer cells and enhanced resistance to DOX [20]. DOX activates the phosphatidylinositol 3-kinase/AKT/mammalian target of the rapamycin signaling cascade and subsequently enhances P-gp expression and promotes the proliferation of cancer cells [82]. DOX also activates the mitogen-activated protein kinase (MAPK)/extracellular signal-regulated kinase pathway, which promotes the proliferation of tumor cells and protects them from oxidative stress [83].

Glucose-regulated protein 78 (GRP78), a chaperone heat shock protein, activates this signaling pathway [84]. Under DOX stress, GRP78 is overexpressed in the cell membrane and induces disordered protein status on membranes, including P-gp. Gemcitabine resistance in breast cancer is associated with overexpressed GRP78 and consecutive AKT elevation, leading to the overexpression of P-gp. It can be interpreted that DOX induces stress in cancer cells, which then overexpress GRP78 and consequently lead to P-gp overexpression, which increases DOX resistance [85]. Colon and prostate cancer cells have shown high GRP78 expression during treatment with celecoxib [86,87]. Collectively, to develop an oral DOX formulation and reduce the occurrence of DOX resistance, the modulation of P-gp function and expression remains a major challenge. Therefore, we also reviewed many DOX formulation studies focusing on the inhibition of P-gp.

#### 3.2.2. P-gp Inhibition in a Cellular Environment to Overcome Drug Resistance

To increase DOX sensitivity, studies have focused on DOX coupled with small molecules or excipients that exert a P-gp inhibitory effect. Grabarnick et al. synthesized PEGylated liposomes incorporating DOX, indocyanine green (ICG), and P-gp inhibitor quinine (ICG + PEGylated liposomes with DOX and quinine [PLDQ]) [88]. ICG is an FDA-approved photosensitizer and is known to be superior to other photosensitizers in terms of tissue penetration and safety [89]. When ICG was exposed to near-infrared light, it generated excessive levels of ROS and caused oxidative stress, leading to ROS-induced cell death [90,91]. When quinine was used as a P-gp inhibitor, PLDQ increased the cellular accumulation of DOX and reduced tumor volume by 25% in mice with xenografted HT29-MDR1 positive cells (i.e., P-gp overexpressed HT29 colon cancer cells) compared with PLD (without quinine) [88]. Additionally, with the incorporation of ICG into PLDQ, the tumor volume of mice with xenografted HT29-MDR1 reduced by 75% following exposure to near-infrared light. The survival rate also showed a 2-fold increase with treatment with ICG + PLDQ. The results could be attributed to the P-gp inhibitory effect of quinine on ICG and DOX as both are substrates for P-gp [88] (Table 3).

An et al. [92] designed DOX-loaded apolipoprotein A1 (ApoA1)-modified cationic liposomes (ApoA1-LipDOX). Previously, ApoA1-modified liposomes increased the intake of the substrate drug by inhibiting the P-gp-mediated efflux [93]. In other words, the DOX concentration in the tumor tissues of 4T1 tumor-bearing mice treated with ApoA1-LipDOX was three times higher than the concentration of free DOX in the same region. Consequently, these mice also had a three times smaller tumor volume [92] (Table 3).

In DOX-resistant H69AR cancer cells, triphenylphosphonium (TPP)-conjugated DOX (TPP–DOX) efficiently accumulates in mitochondria and disrupts the membrane potential and ATP gradient. Consequently, P-gp is inactivated, and mitochondria-induced apoptosis causes cell death [94]. To achieve the targeting of TPP–DOX to tumor mitochondria, Zhou et al. designed a near-infrared (NIR) light- and acidity-activated micellar nanoplatform, known as PEGylated iPUTDN. Then, PEGylated iPUTDN was maintained in circulation. Upon NIR irradiation of the tumor region, PEG was cleaved from the light-sensitive cleavage polymer and 9-amino acid cyclic peptide (cCRGDKGPDC) could facilitate the intratumor penetration and tumor cell uptake of nanoparticles. The acidic condition in tumor cells disrupted the core shell via rapid protonation of poly(β-aminoester)-based nanoparticles and released TPP–DOX from the core. This disrupted the membrane potential and ATP gradient; consequently, P-gp was inactivated, and mitochondria-induced apoptosis caused cell death in the tumor region of H69AR lung cancer-bearing mice. In the tumor region, treatment with PEG-iPUTDN showed a 20-fold greater DOX accumulation than that with free DOX. Compared with TPP–DOX alone, tumor volume decreased by 10-fold following treatment with PEG-iPUTDN and NIR exposure [95] (Table 3).

Suppressing P-gp expression is another strategy to overcome P-gp-mediated DOX resistance. Tomentodione M (TTM), a novel natural syncarpic acid-conjugated monoterpene, increases the intracellular concentration of rhodamine 123 and DOX in K562/MDR human leukemia MDR cells and MCF-7/MDR breast cancer cells [96]. Further, high DOX sensitivity results in cell death when DOX and TTM are used in combination. TTM inhibits the p38 MAPK signaling pathway. P-gp expression stimulated by MAPK is inhibited by TTM, resulting in an increased uptake of DOX in tumor cells [96]. Tanshinone IIA (Tan IIA) is a lipophilic component derived from *Salvia miltiorrhiza*. Tan IIA is a potential candidate for combination with DOX because it not only inhibits DOX efflux but also reduces cardiotoxicity with a cardiovascular protective effect [97,98]. This effect could be explained by the Tan IIA-induced suppression of the PTEN/AKT signaling pathway that downregulates the expression of P-gp as well as BCRP and MRP1 in MCF-7 human breast cancer cells [99]. Collectively, the coadministration of DOX with Tan IIA is a promising candidate for increasing DOX sensitivity and reducing its cardiotoxicity [100].

Ascorbate not only reduces DOX efflux by inhibiting P-gp expression but also sensitizes DOX-resistant MCF-7 breast cancer cells (MCF-7/DOX) to DOX by inhibiting the ATP level [101]. In another attempt to use a combination of DOX and ascorbate, DOX and palmitoyl ascorbate (PA)-loaded liposomes (DOX–PA–liposomes) showed a 2.5-fold higher DOX uptake in MCF-7 cells compared with DOX-loaded liposomes (DOX–liposomes) [102]. Pharmacokinetics and efficacy studies using DOX–PA–liposomes were conducted in SD rats and MCF-7-bearing female BALB/c nude mice for comparison with DOX–liposomes. Intravenous injection of DOX–PA–liposomes in rats showed a 10-fold elevation in DOX AUC compared with that of DOX–liposomes. DOX–PA–liposomes showed a 10-fold lower clearance compared with DOX–liposomes. This indicates that ascorbate reduces extracellular ROS generation and consequently downregulates P-gp expression, resulting in reduced clearance and increased DOX AUC. In addition, the administration of DOX–PA–liposomes in MCF-7-bearing female BALB/c nude mice resulted in a decrease in tumor size by 2-fold compared with that of DOX–liposomes [102].

As the tumor microenvironment is characterized by a low interstitial pH, overexpressed enzymes, and high GSH levels, two silicate nanoparticles (namely glucose oxidase-loaded silica nanoparticles with disulfide bonds in the shell and arginine on the surface [GOD@SiO_2_-Arg] and DOX-loaded MSN [DOX–MSN]) were linked via methacrylated hyaluronic acid (HA–MA) to form hydrogels. Once this formulation was administered to the tumor tissue, hyaluronic acidase, which is overexpressed in tumor tissue, cleaved the crosslink between HA–MA and released GOD@SiO_2_-Arg and DOX–MSN. In tumor cells, Arg generates NO in the presence of H_2_O_2_ and decreases P-gp expression, and the low pH environment facilitates DOX release from DOX–MSN. By combining these tumor microenvironment-responsive formulations, GOD@SiO_2_-Arg and DOX–MSN hydrogel decreased P-gp expression and increased DOX therapeutic efficacy in DOX-resistant MCF-7/ADR cells [103]. In addition, in a nude mouse model of a subcutaneous xenograft tumor, GOD@SiO_2_-Arg and DOX–MSN significantly reduced the tumor volume by 8-fold without causing significant histological abnormalities. The survival rate of tumor-bearing mice in GOD@SiO_2_-Arg and DOX–MSN hydrogel treatment group increased from 15 to 30 days compared with that of DOX treatment group [103] (Table 3).

Pluronic F127 micelles with pH-sensitive polyacrylic acid at two terminals of the micelle carrier (PAA-PF127-PAA-PM) loaded with DOX had a spherical shape with a size of 100 nm. They showed a pH-sensitive DOX release profile, with a faster release at pH 5.0 and a slower release at pH 7.4. PAA-PF127-PAA-PM showed >80% viability at concentrations of <300 μg/mL; moreover, it showed a 3-fold higher apoptosis rate than free DOX in a Walker 256 carcinosarcoma-bearing rat [104]. Pluronic F127 micelles also exhibited a P-gp inhibitory effect [45]. Although the pharmacokinetics and in vivo anticancer efficacy of PAA-PF127-PAA-PM have not been investigated, the pH-sensitive drug release and P-gp reversal effect may have contributed to the therapeutic and pharmacokinetic benefits (Table 3).

#### 3.2.3. Ion-Trapping Phenomenon

Another factor affecting DOX efficacy is the acidic pH of the tumor environment. When ionizable weak base anticancer drugs, such as anthraquinones, anthracyclines, and vinca alkaloids, come into contact with this acidic environment, they become charged, leading to decreased cellular uptake and low therapeutic efficacy. This phenomenon is known as ion-trapping [105,106]. As mentioned previously, the acidity of the central region of solid tumors increases compared with their overall acidity (pH 6.4–7.0). Under this condition, DOX is sequestered into acidic vesicles, reducing the resultant therapeutic efficacy. Treatment with imidazole and tamoxifen, which inhibit vesicle acidification, can increase DOX release into the cytoplasm and enhance its cytotoxicity [105,107,108].

Abumanhal-Masarweh et al. injected NaHCO_3_-loaded HSPC-m2000PEG DSPE–liposomes along with DOX into 4T1 breast cancer-bearing mice. Results showed a 2–3-fold elevation in DOX concentration with NaHCO_3_ coadministration, followed by a decrease in cell viability by 3-fold [109]. The average extracellular pH in tumor tissue increased to 7.38 compared with the acidic extracellular pH of 5.0–6.0 in tumor cells [110].

Ando et al. [111] analyzed per oral NaHCO_3_-loaded liposomes with intravenous Doxil in colon 26 tumor-bearing mice. Both intravenous and orally administered NaHCO_3_ showed clear DOX accumulation in the tumor tissue as well as a 9- and 2-fold decrease in tumor size compared with free DOX and Doxil alone, respectively. A previously reported diffusion model with MDA-MB-231 showed that orally administered bicarbonate had no influence on acidic tumor environment [112]. However, Ando et al. suggested that NaHCO_3_ is a promising candidate for combination with DOX as long as it is released at a local tumor site [111] (Table 3).

#### 3.2.4. Long Noncoding RNA (lncRNA) Overexpression

Recently, lncRNA has been reported to be associated with DOX resistance in osteosarcoma. The expression of LINC00426, a newly found lncRNA, was upregulated in DOX-resistant osteosarcoma [113]. Moreover, following siRNA-mediated LINC00426 knockdown in osteosarcoma cells, the IC_50_ value of DOX decreased by 2-fold, and caspase-3 activity increased by 4-fold [113]. However, the role of lncRNA in DOX resistance is controversial, depending on the cancer type. AX747207, a lncRNA knockdown RUX3 tumor suppressor gene, induces DOX resistance in MCF-7 breast cancer cells [114]. BMP/OP-responsive gene, another lncRNA, induces DOX resistance by activating RPA1 and NF-kB in triple-negative breast cancer [115]. DOX stress induces prosurvival autophagy via lncRNA SOX2OT variant 7, which also results in DOX resistance in osteosarcoma cells. Epigallocatechin gallate in combination with DOX reduces the expression of lncRNA human SOX2 overlapping transcript (SOX2OT) variant 7 in osteosarcoma U20s and SaoS2 cells and reverses the DOX resistance [116].

Additionally, lncRNA SOX2OT variant 7 stimulates upstream Notch3/DLL3 signaling, leading to differentiation of osteosarcoma stem cells as well as breast, lung, and ovarian cancer cells [117]. Consequently, the use of lncRNA inhibitor as a DOX resistance target warrants further research.

#### 3.2.5. Hypoglycemic Environment

Glucose is an important factor in DOX resistance at the cellular level. A hyperglycemic environment activates the mitochondrial function responsible for ROS generation, contributing to the downregulation of P-gp expression and thus increasing DOX sensitivity [118]. Dickkopf protein 4 (DKK4) is an important regulator of glucose uptake; it regulates ROS levels that further regulate P-gp expression [119]. DKK4 mRNA expression was downregulated in hepatoma and colorectal cancer cells (67.5% and 57.1%, respectively) compared with that in normal epithelium [120,121]. Downregulated DKK4 levels are associated with low glucose levels and can contribute to high P-gp expression, which facilitates DOX resistance. In contrast, a study reported the association of DKK4-mediated positive regulation of glucose and ROS and resultant P-gp upregulation; however, the types of cancer cells that upregulate DKK4 levels remain controversial [122].

**Table 3:** Formulations for overcoming DOX resistance in the preclinical stage.

Carrier–Type	Formulation and Route of Administration	Experimental Model	Findings	References
PLs	PL incorporating DOX, ICG, and P-gp inhibitor quinine (ICG + PLDQ), IV	HT-29 MDR1 positive xenograft mice	P-gp inhibition in tumor cells Increased cellular uptake of DOX and reduction in tumor volume by 25% Increase in survival rate by 2-fold	[88]
Liposomes	DOX-loaded apolipoprotein A1-modified cationic liposome (ApoA1-LipDOX), IV	4T1 tumor-bearing mice	P-gp inhibition in tumor cells with 3-fold higher DOX concentration in tumor tissue Decrease in tumor volume by 3-fold	[92]
	DOX and palmitoyl ascorbate (PA)-loaded liposome (DOX-PA-liposome), IV	MCF-7 cells	P-gp inhibition in tumor cells Increased DOX uptake in MCF-7 cells by 2.5-fold	[102]
		SD rats	Elevation in DOX AUC by 10-fold compared with DOX–liposome AUC	
		MCF-7 breast cancer bearing mice	Tumor size decreased by 2-fold compared with DOX–liposome	
	DOX and NaHCO_3_-loaded HSPC-m2000PEG DSPE–liposome, IV	4T1 breast cancer-bearing mouse	Increase in DOX concentration by 2–3-fold The average extracellular pH in tumor tissue increased to 7.38	[109]
	NaHCO_3_-loaded liposome and Doxil combination, IV	colon26 tumor-bearing mouse	Decrease in tumor size by 9- and 2-fold compared with free DOX and Doxil, respectively	[111]
PMs	DOX-loaded Pluronic F127 micelles with pH-sensitive poly(acrylic acid) at two terminals (PAA-PF127-PAA-PM), IV	Walker 256 carcinosarcoma-bearing mice	A 3-fold faster DOX release at pH 5.0 than at pH7.4 A 3-fold higher apoptosis rate compared with free DOX	[104]
PNPs	PEG-iPUTDN + NIR exposure, IP	H69AR lung cancer-bearing mice	Bypassed the P-gp-mediated efflux TPP-conjugated DOX was efficiently accumulated in mitochondria Tumor volume and weight decreased by 10-fold	[95]
MSNs	GOD@SiO_2_-Arg and DOX-MSN hydrogels, SC	MCF-7/ADR cells	Arg generated NO in the presence of H_2_O_2_ and decreased P-gp expression. Low pH facilitated DOX release from DOX–MSN and increased its therapeutic efficacy	[103]
		MCF-7/ADR xenograft mice	Tumor volume reduced significantly by 8-fold without causing significant histological abnormalities Survival rate increased by 2-fold	

PL: PEGylated liposome; PMs: polymeric micelles; PNPs: Polymeric nanoparticles; IV: intravenous injection; IP: intraperitoneal injection; SC: subcutaneous injection; ICG: indocyanine green; GOD@SiO_2_-Arg: glucose oxidase loaded silica nanoparticle with disulfide bonds in the shell and arginine on the surface; DOX-MSN: DOX loaded mesoporous nanosilicate; TPP: triphenylphosphonium; AUC: area under the curve.

### 3.3. Challenges in the Development of Oral Formulations

#### 3.3.1. Low Intestinal Permeability

**Figure 3:**
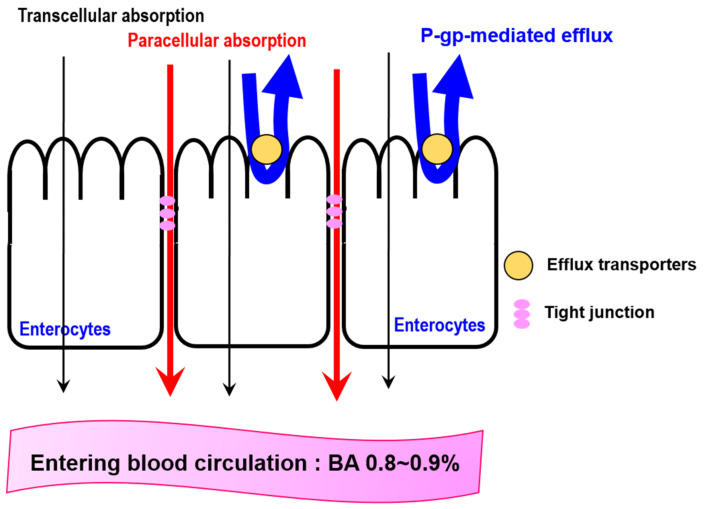
Intestinal absorption of DOX. P-gp: P-glycoprotein. BA: bioavailability, which was accessed in rats [19]. Black, red, and blue arrows represent transcellular absorption, paracellular absorption, and P-gp-mediated efflux, respectively.

Orally administrated DOX encounters a harsh gastrointestinal environment. Among factors inhibiting the BA of DOX, limited intestinal absorption is the most critical factor, with 82–99% of orally administered DOX remaining unabsorbed [19]. Nanoparticles are trapped in the mucus and epithelial barriers. Mucus is mostly composed of glycosylated proteins and is present throughout the gastrointestinal tract [123]. It acts as a first barrier, limiting diffusion and trapping drugs before they interact with the intestinal epithelium [124]. In addition, the epithelium acts as a barrier to drug absorption. DOX also has limited intestinal absorption and P-gp- and MRP1-mediated efflux are the main factors responsible for its low intestinal absorption and BA (Figure 3).

Based on an analysis of bidirectional transport, DOX in Caco-2 cells cultured in a Ca^2+^/Mg^2+^-free medium showed a 20-fold increase in absorptive permeability (P_app,AB_) compared with that in Caco-2 cells cultured in a Ca^2+^/Mg^2+^-positive medium, indicating that DOX is primarily absorbed from the intestinal epithelium through a paracellular route [125]. However, the secretory permeability (P_app,BA_) of DOX was 6.6 times higher than that of P_app,AB_, and the intestinal extraction of DOX via P-gp-mediated efflux accounts for 0.39–0.44 in rats. In the presence of an existing P-gp inhibitor, the P_app,BA_ of DOX reduced significantly, whereas its P_app,AB_ was rarely affected in Caco-2 cells. Collectively, as shown in Figure 3, DOX mainly penetrates the intestinal epithelium via the paracellular pathway, and P-gp-mediated efflux limits DOX absorption. This might be the reason for the low absorption of DOX. Therefore, the oral BA of DOX was 0.8–0.9% [19].

#### 3.3.2. High First-Pass Metabolism of DOX

**Figure 4:**
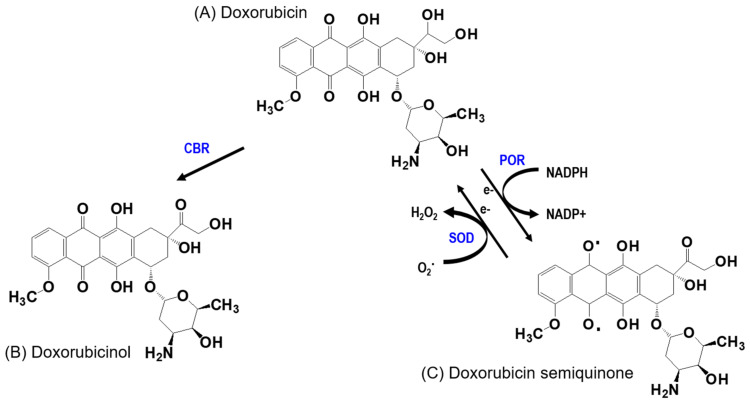
Metabolic pathway and related metabolic enzymes for the transformation of (**A**) DOX to (**B**) doxorubicinol and (**C**) doxorubicin semiquinone, major metabolites of DOX. CBR: carbonyl reductase; POR: cytochrome P450 oxidoreductase; SOD: superoxide dismutase. Arrows represent the metabolic conversion.

In addition to limited intestinal absorption, a previous study revealed that the hepatic first-pass extraction ratio of DOX in rats was 0.49–0.56 [19]. In another study, 45–50% DOX was eliminated via bilirubin excretion in the parent form, and the remaining DOX underwent metabolism [126] (Figure 4). Doxorubicinol (Figure 4B), a major metabolite of DOX-mediated toxicity by carbonyl reductase (CBR), is known to be an essential cardiotoxicity factor that disturbs the homeostasis of iron and calcium balances and induces mitochondrial dysfunction [127,128]. The quinone moiety in DOX is transformed into semiquinone (Figure 4C), another metabolite of DOX, by the cytochrome p450 oxidoreductase (POR) and NADPH dehydrogenase of the mitochondrial electron transport chain complex I [129]. This semiquinone regenerates into quinone and produces ROS. ROS production and cytochrome C, released via mitochondrial dysfunction together, activate caspase-3 and cause cell apoptosis, thus explaining DOX-induced cardiotoxicity [130]. Cardiomyocytes require high levels of ATP; therefore, the density of mitochondria is considerably higher than that of other tissues. Consequently, the heart sustains more damage by DOX than other tissues [131,132].

### 3.4. Formulation Strategy for Overcoming Low Oral BA in the Preclinical Stage

#### 3.4.1. Coadministration with a P-gp Inhibitor to Increase Oral Absorption

Two cytosolic ATP-binding cassette (ABC) domains of a P-gp inhibitor and ATP hydrolysis alter the conformation of P-gp and allow the excretion of substrate drugs into the extracellular environment. Because the ABC domains and substrate binding sites are available for targeting with small molecules, to date, various P-gp inhibitors have been developed to downregulate the expression and activity of P-gp [133]. As described previously, the use of P-gp inhibitors is critical for achieving acceptable oral BA and preventing DOX resistance at the cellular level. Many research studies have focused on this issue.

The concomitant administration of a P-gp inhibitor with anticancer drugs as a strategy for BA enhancement has been studied in clinical trials. Elacridar (GF120918) is one of the third-generation P-gp and BCRP inhibitors that more specifically inhibits P-gp and BCRP while having no interaction with CYP enzymes [134]. Elacridar is a noncompetitive P-gp inhibitor and modulates ATPase activity by inhibiting ATP hydrolysis [135]. With the coadministration of elacridar, the oral BA of paclitaxel increased from 4% to 30–50% in humans [136,137]. Topotecan also showed a marked BA enhancement. With the coadministration of elacridar, the oral BA of topotecan increased from 42% to 97% in cancer patients. In other studies, interindividual variability decreased from 17% to 11% [138,139,140]. Similar clinical trials using potent P-gp inhibitors such as encequidar (HM30181A) with paclitaxel and docetaxel are underway [141]. Coadministration of the P-gp inhibitor ONT-093 with docetaxel resulted in BA enhancement from >10% to 26% and lowered interindividual variability from 90% to 44–70% [142].

A clinical trial explored DOX administration in combination with elacridar via IV injection in 46 patients. The DOX AUC tended to increase with increasing plasma elacridar concentrations, but only a small difference in AUC was observed between treatment with DOX alone and the combination of DOX and elacridar [126]. However, the plasma concentrations of doxorubicinol increased in some patients, which may be attributed to the decreased metabolism of doxorubicinol owing to the presence of elacridar. Similar results were reported in combination treatment with DOX and cyclosporin A or PSC-833 [143,144]. Therefore, unlike paclitaxel, the coadministration of elacridar with DOX may not provide the therapeutic benefit of DOX.

Zosuquidar (LY335979) is the most selective third-generation P-gp inhibitor and has no interaction with efflux transporters, such as BCRP and MRP transporters, or CYP. In UKFNB-3 neuroblastoma cells, DOX treatment with zosuquidar showed a 2-fold lower IC_50_ value than DOX alone [145]. With the coadministration of zosuquidar, Nielsen et al. reported a 2.5–35% increase in the oral BA of etoposide in rats [146]. However, currently, clinical trials based on the combination of zosuquidar and the CHOP regimen, which includes the intravenous infusion of vincristine (1.4 mg/m^2^), DOX (50 mg/m^2^) and cyclophosphamide (750 mg/m^2^) and the oral administration of prednisolone (100 mg), have not revealed positive interactions between zosuquidar and P-gp substrates in the CHOP regimen (i.e., vincristine or DOX) [147].

As studies have reported a substantial contribution of P-gp to intestinal absorption, it is necessary to conduct clinical studies to investigate the effect of P-gp inhibitors on the oral BA of DOX. In addition, the effect of P-gp inhibitors on the oral BA of anticancer drugs is likely to benefit only certain drugs, especially those with highly variable drug concentrations, low oral BA, and anticancer activity primarily mediated by the parent drug with a demonstrated exposure–response relationship [58]. Some drugs such as etoposide did not significantly improve their oral BA despite promising preclinical evidence [38,148]. Therefore, additional functional inhibitors that can increase intestinal penetration as parent drugs are required for formulating DOX with P-gp inhibitors.

#### 3.4.2. Mucoadhesive Formulation

Adhesion to and penetration across the thick mucosa in the gastrointestinal tract is an important aspect of oral administration, which has been a continuing concern in the development of oral therapies. In this regard, chitosan is a cationic polysaccharide derived from chitin that has been widely used because of its mucoadhesive property and ability to loosen the epithelial tight junction in the gastrointestinal tract, making it an important conjugate candidate for oral formulation [149,150]. Chitosan and stearic acid copolymer (CSO–SA) were used to form mixed micelles with a diameter of 32.7 nm in the aqueous phase, which were subsequently uptaken by cancer cells. DOX-conjugated CSO–SA PMs (DOX–CSO–SA) enhanced DOX uptake in MCF-7/ADR cells and human hepatocellular carcinoma-bearing nude mice. This formulation showed favorable drug release at an acidic pH (pH 5.0) compared with that at pH 7.0. DOX–CSO–SA micelles were sensitive in both DOX-sensitive MCF cells and DOX-resistant MCF-7/ADR cells. The reversal power, which was calculated based on the IC_50_ difference, against MCF-7/ADR cells was 10.5 [151]. Similar approaches have been applied to oral administration. Compared with free DOX, chitosan and linoleic acid-based PMs incorporating DOX (DOX–CS–LA) improved the oral BA of DOX by 166% in Sprague-Dawley (SD) rats by targeting the intestinal fatty acid transporter [152].

With the increasing development of multifunctional formulations, the addition of P-gp inhibitors and chitosan into the DOX formulation could increase its oral BA. Therefore, we examined DOX formulation studies that aimed to increase the oral BA of DOX.

#### 3.4.3. Formulations to Increase the Oral BA of DOX

In addition to the DOX formulation in the clinical stage, many DOX formulations, such as PMs, PNPs, etc., that are mainly studied for increasing oral BA, targeting the tumor cells, and reducing adverse effects have been studied.

(1) PMs

In a previous study, DOX-loaded lysine-linked ditocopherol polyethylene glycol 2000 succinate micelle formulation (PLV2K–DOX) increased the intestinal absorption rate of DOX by 1.61–3.19-fold compared with free DOX in the duodenum, jejunum, and ileum [104]. In the presence of cyclosporine, a P-gp inhibitor, the cellular uptake of DOX was even higher than that with free DOX, suggesting that the increased intestinal permeability of DOX incorporated in PLV2K–DOX is attributed to P-gp inhibition by the ditocopherol polyethylene glycol 2000 succinate linkage. In addition, caveolin-mediated and caveolin-/clathrin-independent endocytosis facilitated the intestinal absorption of DOX. The pharmacokinetics of DOX in rats following oral administration of PLV2K–DOX revealed a 3.7- and 5.6-fold higher maximum plasma concentration (C_max_) and plasma exposure (AUC), respectively, than those of free DOX [153,154] (Table 4).

Oleanolic acid (OA) is a naturally occurring pentacyclic triterpenoid saponin present in >1600 plant species [155]. It exerts hepatoprotective, anticancer, anti-inflammatory, and antioxidative effects. OA also induces ROS generation, apoptosis, and cell cycle arrest and attenuates DOX-mediated toxicity in patients with hepatocellular carcinoma [156]. Based on this effect, Kumbham et al. [155] developed a biodegradable micelle formulation encapsulating DOX and OA. The OA-conjugated methoxy-poly (ethylene glycol) (mPEG)-polylactide (PLA) micelle formulation loaded with DOX (mPEG–PLA–OA–DOX) enhanced DOX accumulation, increased cell cytotoxicity, and induced apoptotic signals. This formulation also increased DOX accumulation and antitumor activity in FaDu-HTB-43 spheroids isolated from a hypopharyngeal tumor of a patient with squamous cell carcinoma. The formulation showed a 30-fold enhancement in circulation time and a 30-fold reduction in the clearance time of DOX compared with free DOX following their oral administration in rats (Table 4).

(2) PNPs

Among the PNPs presented in Section 2, PLGA has been approved by the US FDA for encapsulating various drugs to achieve ease of administration, biocompatibility, and biodegradability [157,158]. Moreover, PLGA can be uptaken by M cells distributed in the Peyer’s patches of the small intestinal epithelium for distribution in the lymphatic circulation. This route is crucial, as it can bypass the first-pass mechanism and P-gp-mediated efflux in the intestinal epithelium [159,160,161]. Biodegradable nanoparticles containing DOX–PLGA have been developed for treating glioblastoma and breast cancer in animal models, but they have not yet undergone clinical studies [162,163,164].

Several DOX formulation studies in experimental animals showing enhanced oral BA and therapeutic efficacy have been reported. The preparation of freeze–dried DOX-loaded PLGA nanoparticles resulted in a 3.63-fold BA enhancement, while its absorption time (T_max_) was delayed from 6 to 36 h. The sustained release of DOX from freeze–dried DOX-loaded PLGA NPs also has the advantage of not causing cardiotoxicity [157]. Treatment with DOX-loaded PLGA NPs showed considerably greater cellular accumulation than that with free DOX and even greater cellular accumulation than that with free DOX and cyclosporine A. Orally administered DOX-loaded PLGA NPs showed a similar reduction in tumor size and burden to that of IV administered DOX, whereas oral free DOX was shown to be ineffective. This formulation increased the survival rate of breast tumor-bearing female rats compared with free DOX. Moreover, the increased levels of well-known cardiotoxicity markers—malondialdehyde, lactate dehydrogenase, and creatine phosphokinase—reduced with the decreasing levels of GSH [157,165]. Compared with elevated or reduced levels of markers in IV DOX, superoxide dismutase levels remained unchanged in DOX-loaded PLGA nanoparticles. Surface modification of PLGA NPs using Pluronic F127, zwitter ionic polydopamine, and PEGylation increased the mucus and epithelial permeability of DOX and showed a great improvement in its oral BA [166]. These three surfaced-modified PLGA NPs showed significantly increased mucus and epithelial penetration, cellular uptake, and transepithelial transport in HT29-MTX and TR146 cells. The in vivo effect of these surface-modified PLGA NPs has been evaluated (Table 4).

PEGylated DOX-loaded PLGA NPs were prepared to circumvent the effect of intestinal efflux transporters. This formulation had a size of approximately 200 nm and a zeta potential of −13.1 mV. It showed sustained release for 24 h. The plasma AUC of DOX was 13.8 times higher than that of free DOX, with a 2.1-fold delayed elimination rate. It also showed enhanced intestinal adhesion and penetration compared with non-PEGylated particles [167]. Another surface modification of PLGA NPs has been reported. Chitosan-coated daunorubicin–PLGA (Cs–DAU–PLGA) nanoparticles had the following characteristics: (1) biodegradability and biocompatibility owing to PLGA and (2) a mucoadhesive property and ability to open a tight junction owing to chitosan. The properties of controlled release were attributed to the formulated nanoparticles. These properties were demonstrated by the 3.5-fold increase in Caco-2 permeability and endocytotic intestinal uptake of the Cs–DAU–PLGA nanoparticle formulation, which were not observed with free dauonorubicin. Compared with free daunorubicin, the administration of Cs–DAU–PLGA nanoparticle formulation to rats revealed an 11.3-fold higher AUC with a 2.8-fold delay in elimination half-life. The enhanced absorption and delayed disposition could be due to encapsulation with daunorubicin, escape from P-gp-mediated efflux and CYP-mediated metabolism, and enterocytic endocytosis of the nanoparticle formulation [168] (Table 4).

As acidic conditions (pH < 6.0) are required to dissolve chitosan in an aqueous solution [169], acidic modification of chitosan has been attempted. Chitosan was modified to chitosan diacetate and chitosan triacetate, and DOX-loaded nanoparticles were prepared using modified chitosan diacetate (DOX–CDA) or modified chitosan triacetate (DOX–CTA) with loading efficacy of 58% and 80%, respectively. Both modified chitosan–DOX-loaded NPs showed a 2.1- and 1.8-fold increase in permeability in MCF-7 tumor cells compared with free DOX. DOX–CTA NPs showed a relatively sustained release of DOX over 24 h, and the oral administration of DOX–CTA NPs in rats showed a 3-fold AUC enhancement compared with that of free DOX [169] (Table 4).

Intestine-penetrating, pH-sensitive, and double-layered NPs with a mean size of 350 nm were developed. Hydrophobic polyortho-ester urethane, composed of PCL and polyoxyethylene (POE) blocks, constituted the core shell, and DOX was loaded into the core shell. Carboxymethyl chitosan and glutaraldehyde were crosslinked to the outer membranes. The outer coating of carboxymethyl chitosan loosened the tight junction of the intestinal epithelium, and glutaraldehyde stabilized the liposome in the harsh gastric environment (pH 0.9–1.5) without releasing DOX; therefore, it could bypass the first-pass effect of DOX [170]. The core POE block induced DOX accumulation at the tumor site and DOX release in the acidic tumor environment (pH 5–6). PO administration of these formulations in H22 tumor-bearing mice showed a relative BA of 75.4%, which effectively inhibited tumor growth. Importantly, orally administered intestine-penetrating, pH-sensitive, and double-layered DOX NPs showed reduced cardiac distribution compared with free DOX, and the DOX concentrations in the major tissues did not exceed the maximum tolerated concentration; approximately 40% of absorbed DOX was accumulated in tumor tissues [170] (Table 4).

The natural substance casein has become a candidate for anticancer formulations because of its advantages, such as low cost, biodegradability, nontoxicity, and ability to form nanomicelles and nanoparticles [171]. Sodium caseinate NPs incorporating DOX (DOX–NaCN), with a size of 271 nm, spherical shape, and zeta potential of −0.054 mV, have been prepared and characterized. They showed sustained DOX release over 24 h and a significantly higher cellular uptake. Orally administered DOX–NaCN decreased the tumor size by 8-fold compared with free DOX in 4T1-breast cancer-bearing mice. In addition, the oral administration of DOX–NaCN showed 8.34-fold higher DOX accumulation in tumor tissues than that of intravenous free DOX but was comparable to that of intravenous DOX–NaCN. However, the cardiac concentration of DOX following the oral administration of DOX–NaCN was the lowest among the four different treatment groups (i.e., free DOX administered orally or intravenously, DOX–NaCN administered orally or intravenously). These results suggest that nontoxic controlled release of DOX from NaCN has beneficial antitumor effects after PO administration [172] (Table 4).

(3) Multilayer micro-dispersing system (MMS)

Feng et al. [173] constructed MMS to enhance the oral BA of DOX. First, nanogels (NGs) incorporating DOX were constructed with chitosan to obtain a carboxymethyl chitosan complex (DOX:CS/CMCS-NGs), which was then crosslinked with Ca and carboxylate ions in the core of multilayer alginate beads. These beads were composed of a layer-by-layer structure with a porous core, along with quercetin (DOX:CS/CMCS-NGs/Qu-M-ALG-beads). At low pH of 7.0, DOX:CS/CMCS-NGs/Qu-M-ALG-beads resisted the swallowing test, but DOX:CS/CMCS-NGs and quercetin were gradually released at pH > 7.0. Chitosan induced mucoadhesion and, more importantly, the ability to open the tight junction in the intestinal epithelium, which promoted DOX paracellular permeation [149,150]. Quercetin, a P-gp inhibitor, enhanced DOX absorption by inhibiting the P-gp-mediated efflux of DOX. In addition, the M-cell-mediated endocytosis of DOX:CS/CMCS-NGs could increase the BA of DOX. As a result, orally administered DOX:CS/CMCS-NGs/Qu-M-ALG-beads had a 18.65-fold higher AUC compared with free oral DOX, and its absolute BA was calculated as 55.8% [173] (Table 4).

(4) MSNs

MSNs have been approved by the US FDA for clinical trials of cancer formulations because of their adjustable porous structure, ability to induce surface modification, high loading efficiency, excellent biocompatibility, and biodegradability [174]. The pharmacokinetics of DOX-loaded MSNs of three different sizes or shapes were evaluated in rats. The particle size of MSNs ranged from 100 to 200 nm with a stable negative zeta potential. The viability test in Caco-2 cells revealed that 80% of MSNs were nontoxic. DOX–MSN with a rod shape and size of 200 nm had higher C_max_ and greater AUC than orally administered free DOX. The relative BA enhancement of DOX–MSN with a rod shape was 5.9-fold compared with free DOX [175] (Table 4).

With the ease of surface modification in MSNs, DOX loading and surface functionalization of MSNs to modify their release profile and therapeutic efficacy have also been investigated. DOX-loaded MSNs modified with (3-aminopropyl)triethoxysilane (DOX–MSN–APTES) possess a negative charge under normal cell conditions (pH 7.4), which becomes positive after exposure to the acidic tumor environment (pH 5.0). The benefit of charge-reversible MSNs is long-term drug stability in the serum (pH 7.4), which permits the sustained release of DOX for 7 days in KB cells; in surface unmodified MSNs, DOX release was completed within 8 h [176].

Furthermore, DOX–MSN coated with soybean lecithin and DSPE-PEG2000 (DOX–MSN–phospholipids) have been formulated. DOX–MSN coated with phospholipids increased the zeta potential from −25 to −1.0 mV and enhanced the affinity toward the cell membrane lipid bilayer. Consequently, DOX–MSN–phospholipids showed a pH-sensitive release profile (i.e., 3.5–5-fold higher DOX release at pH 5.0 than at pH 7.4) and enhanced internalization of DOX–MSN–phospholipids. Despite the relatively low loading efficiency of 16%, DOX–MSN–phospholipids showed a 2-fold increase in cytotoxicity and 10-fold reduction in hemolysis percentage [177].

Dual-stimuli-responsive HA conjugated with MSN via a disulfide link was prepared. CD44 receptors were responsive to HA and actively uptook HA-modified MSNs encapsulating DOX, which resulted in 3-fold higher DOX uptake in CD44-positive HCT-116 cells than that in CD44-negative NIH-3T3 cells. Another study used GSH as a stimulant. High levels of GSH facilitated enhanced DOX release at low pH (pH 5.0) compared with that at pH 7.4 [178]. This surface modified MSN could be a strategy for stimuli-responsive targeted cancer therapy.

(5) Clay mineral formulation

Clay minerals are biocompatible and low-cost materials that have been shown to modify the release and increase the solubility of drugs [179]. Recently, hematite NPs were added to DOX loaded chitosan-poly vinyl pyrrolidone hydrogels to deliver DOX to MCF-7 cancer cells, based on its pH-responsiveness. This formulation enabled pH-sensitive delivery to cancer cells and sustained DOX release [180]. Montmorillonite (MMT) clay mineral has been frequently used as a drug carrier due to its excellent cation exchange capacity and biocompatibility. Rahmani et al. [181] prepared a pH—sensitive chitosan—MMT—nitrogen—doped carbon quantum dots (NCQDs) nanocomposite and loaded DOX. This formulation showed pH-sensitive sustained release of DOX at pH 5.4 over a 96-h period, but no diffusion was observed at pH 7.4. It also showed significantly higher cytotoxicity toward MCF-7 cells compared with free DOX [181]. In addition, MMT nanosheets effectively intercalated and stabilized DOX. MMT also showed pH-sensitive sustained release at pH 6.0 and increased cytotoxicity in MCF-7 cells. pH-sensitive release profiles of DOX from MMT nanosheets are related to the protonation of negatively charged nanoclays in weakly acidic solutions, which make it easier to dissociate with positively charged DOX [179,182].

Similarly, Huang et al. prepared four layers of MMT nanosheets that stably intercalated PEGylated chitosan (PEG-CS/MMT). The multilayered PEG-CS/MMT showed superior DOX loading efficiency, was located within acid organelles, and elicited cell apoptosis [183], which can give a rationale to MMT nanosheets as a cancer chemotherapeutic drug delivery system. Further investigations regarding the beneficial effects on the pharmacokinetics and therapeutic effects of DOX in in vivo preclinical and clinical studies need to be performed.

**Table 4:** DOX formulations to increase oral BA.

Carrier–Type	Formulation & Route of Administration	Experimental Model	Findings	References
PMs	Linolenic acid–chitosan-based PMs (DOX-CS-LA), PO	SD rat	Mucoadhesive formulation Targeting the intestinal fatty acid transporter Increase in relative BA by 166% compared with that of free DOX	[152]
	Lysine-linked ditocopherol polyethylene glycol 2000 succinate (PLV2K-DOX), PO	SD rat	Intestinal permeability of PLV2K–DOX was 3.19-, 1.61-, and 1.80-fold higher than that of free DOX in the duodenum, jejunum, and ileum Orally administered PLV2K–DOX showed 5.6-fold higher AUC than free DOX in rats	[153,154]
	Oleanolic acid conjugated methoxy-poly (ethylene glycol)-poly (D, L-lactide) (mPEG-PLA-OA), PO	Wistar rats	A 30-fold increased DOX circulation time and 30-fold reduced clearance time	[155]
PNPs	DOX-loaded poly (lactic-co-glycolic acid) (PLGA) NPs, PO	SD rats	BA enhancement by 363% and reduced cardiotoxicity	[165]
		Breast cancer bearing rats	Reduced tumor size, increased survival rate, and reduced cardiotoxicity	
	Chitosan coated–daunorubicin PLGA–NPs, PO	Wistar rats	Compared with free daunorubicin, a 11.3-fold higher AUC and 2.8-fold delay in the elimination of daunorubicin from the plasma	[168]
	PEGylated-DOX-loaded-PLGA–NPs, PO	Wistar rats	Compared with free DOX, a 11.8-fold higher AUC and 2.1-fold delay in the elimination of DOX from the plasma	[167]
	Chitosan modified chitosan diacetate (CDA) and chitosan triacetate (CTA)-NPs, PO	MCF-7 cells	Approximately 2-fold increased permeability of DOX in MCF-7 cells	[169]
		SD rats	Compared with free DOX, sustained release for 24 h, and 3-fold increase in the AUC of DOX–CTA NPs	
	Intestine-penetrating, pH-sensitive and double layered NPs, PO	H22-tumor bearing mice	Relative BA of 75.4% with effective inhibition of tumor growth DOX concentrations in major tissues did not exceed the maximum tolerated concentration Approximately 40% of the absorbed DOX accumulated in the tumor tissue	[170]
	Sodium caseinate (NaCN) NPs, PO	4T1-breast cancer bearing mice	A 8-fold tumor shrinkage compared with that of free DOX Following the oral administration of DOX–NaCN NPs, DOX in tumor tissues showed 8.34-fold higher accumulation than IV DOX and 1.27-fold higher accumulation than IV DOX–NaCN NPs	[172]
MMS	Mutilayer alginate beads with codelivery of chitosan-DOX nanogel and quercetin (DOX:CS/CMCS-NGs /Qu-M-ALG-Beads), PO	SD rats	pH-sensitive release at pH > 7.0. Chitosan increased DOX absorption via mucoadhesion and tight-junction opening Quercetin increased DOX absorption by inhibiting P-gp. BA of DOX:CS /CMCS-NGs/Qu-M-ALG-beads was 55.8%	[173]
MSNs	DOX loaded MSN (DOX-MSN), PO	SD rats	DOX–MSN with a rod shape and size of 200 nm showed 5.9-fold enhancement in relative BA compared with free DOX	[175]

PM: Polymeric micelle; PNP: Polymeric nanoparticles; MMS: Multilayer micro-dispersing system; MSN: Mesoporous silica nanoparticles; DOX:CS/CMCS-NGs/Qu-M-ALG-Beads: DOX-chitosan complex incorporating carboxymethyl chitosan nanogels in the core of MMS and querctin modified alginate beads; AUC: Area under the curve; PO: per os.; BA: Bioavailability; SD rats: Sprague-Dawley rats.

## 4. Future Perspectives

IV administrations of liposomal DOX formulations have shown great improvement in terms of prolonged DOX circulation and reduced cardiotoxicity. Future DOX formulation strategies can be developed via three approaches: (1) increasing tumor targetability using the tumor microenvironment, (2) increasing therapeutic efficacy by achieving more favorable pharmacokinetic properties and reducing DOX resistance, and (3) enhancing the oral BA by switching from IV to PO formulation. DOX formulations that inhibit the P-gp function have been evaluated for developing more effective formulations that can reduce the occurrence of drug resistance and enhance oral absorption. However, the use of a simple P-gp inhibitor in DOX formulations seemed to be ineffective. In addition, pH- or ROS-sensitive DOX formulations (e.g., pH-sensitive PLs, PNPs, and PMs and ROS-sensitive liposomes and MSNs; Table 2) effectively increased DOX concentrations in tumor cells following parenteral administration. In particular, the use of ROS- or pH-sensitive excipients along with Rc-targeted ligands in the outer shell and DOX and P-gp inhibitor inside of core formulation have been reported to increase the targetability of the formulation and sequential release of DOX and P-gp inhibitors in tumor cells. Consequently, these DOX formulations reduced the tumor size (Table 3); however, they were also administered parenterally.

Regarding the oral formulation of DOX, multifunctional and sequential release of functional excipients may show promising BA enhancement and more effective anticancer activity. DOX undergoes limited intestinal absorption because of low paracellular permeability and P-gp-mediated efflux. To increase the intestinal absorption of DOX, a mucoadhesive agent, tight-junction modulator, and/or P-gp inhibitor must be used as the outer shell of the formulation. After absorption, DOX formulations with a pH- or ROS-sensitive core can show better tumor targetability and provide therapeutic benefits. Among the tested formulations, chitosan-modified or intestine-penetrating, pH-sensitive, and double-layered nanoparticles (PNP and MMS; Table 4) significantly increased oral BA, showed enhanced antitumor activity, and reduced cardiotoxicity [170]. Nevertheless, these oral DOX formulations have not yet been tested on humans.

These multifunctional and sequential-release DOX formulations warrant further validation in patients with cancer, and the success of these formulations will depend not only on improved efficacy, reduced toxicity, and enhanced oral BA in humans but also on improved manufacturing processes and market competition. We hope that this strategy of creating multifunctional and sequential-release DOX formulations using a mucoadhesive agent, tight-junction modulator, and P-gp inhibitor in the outer shell and a pH- or ROS-sensitive core will expand the oral administration of DOX.

## Data Availability

Not applicable.
